# Neck Cooling Improves Table Tennis Performance amongst Young National Level Players

**DOI:** 10.3390/sports5010019

**Published:** 2017-03-11

**Authors:** Terun Desai, Lindsay Bottoms

**Affiliations:** 1 School of Health, Sport and Bioscience, University of East London, Stratford, London E15 4LZ, UK; t.desai@herts.ac.uk; 2 Department of Psychology and Sports Science, University of Hertfordshire, Hatfield AL10 9AB, UK

**Keywords:** skill, fatigue racquet sports, ice, thermoregulation

## Abstract

This study aimed to examine the effects of neck cooling on table tennis performance. Eight young, National level, male table tennis players (age 16 ± 2 years, height 1.77 ± 0.08 m, body mass 67.54 ± 10.66 kg) were recruited. Participants attended four testing sessions separated by a week. Session one determined fitness levels, and session two was a familiarisation trial. The final two sessions involved completing the table tennis-specific protocol either with (ICE) or without (CON) neck cooling for 1 min before each exercise period (bout: 80–90 shots), which represented an individual game. The exercise protocol required completing three bouts to represent a match, each simulating a different skill (forehand, backhand, alternate forehand and backhand), against a mechanical ball thrower. Performance was measured by the number of balls hitting two pre-determined targets. Heart rate, ratings of perceived exertion (RPE), and thermal sensation (TS) were measured. Total performance scores (shots on target) were significantly greater during ICE (136 ± 26), compared to CON (120 ± 25; *p* = 0.006) with a 15 (±12)% improvement. Effects for time (*p* < 0.05) but not condition (*p* > 0.05) were found for RPE and all other physiological variables. TS significantly decreased with cooling throughout the protocol (*p* = 0.03). Neck cooling appears to be beneficial for table tennis performance by lowering thermal sensation.

## 1. Introduction

Table tennis is an intermittent sport with periods of high-intensity exercise, requiring high anaerobic endurance, interspersed with short rest intervals [1]. International tournaments are commonly played in arenas with central ambient temperature control, therefore temperatures are kept constant throughout competitions. Multiple same-day competitions are commonplace in table tennis tournaments, therefore, performance impairments due to thermal stress are common [2]. Consequently, thermoregulation becomes important to maintain normal body temperature and prevent fatigue impairing performance through physiological or psychological pathways [3].

Attention has shifted towards the psychological effects of thermoregulation in terms of thermal sensation as opposed to the physiological changes that are normally described when elucidating its effects on sports performance [4]. The central governor is critical to both physiological and psychological pathways, where the central governor theory dictates that the brain as a master regulator, maintains homeostasis by eliciting effector responses in reaction to exercise-induced fatigue, based on physiological and psychological afferent feedback such as increased temperature [5]. In terms of affecting thermoregulation during exercise in hot environments, attaining critical core temperature (T_c_) induces fatigue and therefore prevents exercise at the intensity eliciting peak performance [6,7]. Physiologically, the central governor theory suggests that an athlete’s brain anticipates reaching critical core temperature and alters exercise intensity to avoid reaching such a temperature, thus allowing the athlete to continue exercising, albeit at a lower intensity [8,9]. Consequently, the implications of not exercising at the intensity eliciting peak performance negatively impacts skill-based sports [10]. Therefore, cooling could improve table tennis performance by facilitating players to perform at the intensity eliciting peak performance through reducing the perception of physical exertion.

There are several methods for cooling which are predominantly encompassed by pre-cooling and per-cooling methods using different modalities, thus cooling as an intervention to improve performance has produced mixed findings [11]. The type and duration of application of the cooling modality varies between sports [4], subsequently making comparisons on the effectiveness of cooling between these difficult. The optimal cooling strategy is dependent on the dynamics of the sport, where intermittent sports with regular intervals permit both pre- and per-cooling, thus maintaining lower thermal stress throughout the duration of the exercise [12]. Being an intermittent sport, application of cooling interventions before match play, between games, and during tactical timeouts may improve table tennis performance.

Performance improvements with cooling are often a result of physiological changes such as reducing core temperature and increasing heat storage capacity [11]. However, studies incorporating pre- and per-cooling strategies have reported reduced thermal load and ratings of perceived exertion (RPE) during exercise in the heat with concurrent improvements in performance [13,14]. In recent years, authors have focused on the psychological effects of neck cooling, through the application of a cooling collar, which dampened perceived thermal sensation resulting in increased time to volitional exhaustion during fixed-intensity running [15,16]. Additionally, the effects of non-thermal neck cooling, predominantly from menthol sprays, on thermoregulation and performance suggest that improvements are conferred via preferable changes in RPE and thermal sensation rather than alterations in T_c_, baseline skin temperature (T_sk_), skin blood flow, and sweat rate [17], lending further support to the psychological implications of cooling. In a sport such as table tennis which has a short exercise duration and short breaks between play, athletes could benefit from the easy application of neck cooling by reducing RPE and thermal strain whilst maintaining skill. Therefore, improvements in performance could be through psychological as opposed to physiological alterations due to the intermittent nature of table tennis allowing adequate recovery time between games and matches. This study therefore aimed to examine the effect of cooling on table tennis performance, in particular shot accuracy, as it has not been previously researched. It was hypothesised that intermittent cooling may improve table tennis performance by reducing perceived exertion and thermal sensation.

## 2. Materials and Methods

### 2.1. Participants

Eight young, National level, male table tennis players (age 16 ± 2 years (age range 15–21 years), height 1.77 ± 0.08 m, body mass 67.54 ± 10.66 kg, $\dot{V}$O_2peak_ 49.8 ± 5.9 mL·kg^−1^·min^−1^, competitive playing experience 6 ± 3 years) of international and national playing standard were recruited from a table tennis club. No attempt was made to categorise the players based on biological age. All participants reported table tennis training at least twice weekly and playing in competitions at least twice monthly and were injury free at the time of testing. Data collection occurred between March and July 2015. All participants completed informed assent forms and under 18s also completed parental consent forms. School of Health, Sport and Bioscience Ethics Committee at the University of East London gave ethical approval for the study’s experimental procedures, which followed the principles outlined in the Declaration of Helsinki.

### 2.2. Protocol

**Figure 1:**
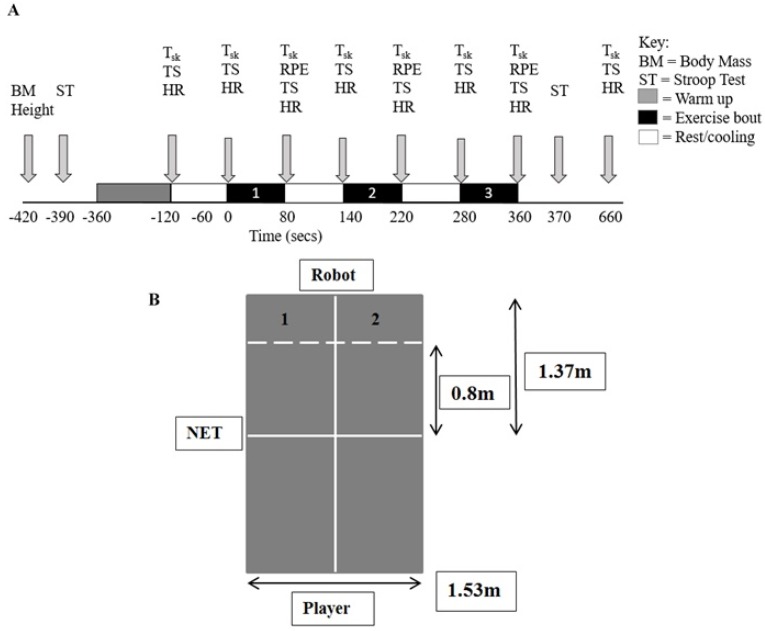
(**A**) Schematic of testing protocol. (**B**) Illustration of table and target setup for exercise protocol. T_sk_, baseline skin temperature; TS, thermal sensation scale, HR, heart rate; RPE, ratings of perceived exertion.

A counterbalanced, within-group test design was used, where participants were their own control measure. Participants attended four sessions; the first was an incremental exercise test to exhaustion on a treadmill to determine their $\dot{V}$O_2peak_, the second was a familiarisation session which replicated the main experimental trial (without cooling) to orientate themselves with the equipment and protocol, thus reducing learning effects. The final two visits were the main experimental trials which were all performed in the afternoon and separated by 7 days. Participants were randomly assigned into two groups. The first group conducted the control test (non-cooling (CON)) whilst the second group conducted the experimental test (cooling (ICE)) during their first data collection visits. Groups then swapped tests for the second data collection visit, thereby minimising any order effect [13]. The cooling method comprised of an ice-bag, with a combined thickness of 1.2 µm (Pull-N-Pak Xtreme, Crown Poly, Huntington Park, CA, USA), filled with 310 g [18] of cubed ice and kept between 0 and 3 °C inside a cooler. The present study respected ecological validity by using an ice-bag, as it is cost-effective, simple, portable, and has been demonstrated to improve repetitive anaerobic performance [19], and is therefore ideal for improving table tennis performance. Cooling was applied to the neck rather than the major muscles used in table tennis, as cooling impairs neuromuscular function [20] and has been found to alleviate thermal strain [15]. It was applied three times in each trial directly on the nape of the neck for 1 minute pre-exercise and after the first and second bout, as this is the time allowed between games and during tactical timeouts in table tennis. To standardise testing, participants fasted for 2 hours prior to each session and drank 500 mL water 60 minutes before all testing sessions to ensure they were euhydrated [21]. For the main experimental trials, baseline measures were recorded followed by the Stroop test and then participants each completed a 4-minute table tennis-specific warm up. Participants completed three exercise bouts to simulate match play conditions with a minute rest (passive standing) incorporated between bouts in order to simulate intervals between games and allow measurements to be taken. During the bouts, participants had to play 80 shots for the forehand and alternate forehand/backhand bouts and 90 shots for the backhand only bout whilst aiming to hit pre-determined targets. Following the exercise, participants completed another Stroop test. The protocol can be seen in Figure 1A.

### 2.3. Procedures

#### 2.3.1. Incremental Exercise Test to Exhaustion

During the first visit, participants completed a $\dot{V}$O_2max_ test. Prior to the test, participants’ age, height (Seca Leicester Stadiometer, Seca, Birmingham, UK), and body mass (Tanita Body Composition Analyser BC-418MA, Tanita Corporation, Tokyo, Japan) were recorded. The test was carried out on a treadmill (HP Cosmos, h/p/cosmos sports and medical GmbH, Munich, Germany) where heart rate (HR) (Polar T31c and FT1, Polar Electro, Kempele, Finland) and gas analysis (Metalyser 3BR2, Cortex Biophysik GmbH, Leipzig, Germany) were recorded throughout to determine $\dot{V}$O_2peak_. The participants started at 6 km·h^−1^ at 1% gradient for one minute and speed increased 1 km·h^−1^ every minute until volitional exhaustion.

#### 2.3.2. Testing Procedure for Experimental Trials

Trials were conducted at a dry-bulb environmental temperature and humidity of 21.3 ± 3.4 °C (Data-Therm IR JXB-182, Guangzhou Jinxinbao Electronic Co. Ltd, Guangzhou, China) and 44.5% ± 4.0%, respectively, and were measured before each test. Participants’ body mass (kg) was measured pre-exercise, followed by baseline skin temperature (T_sk_), HR, and a nine-point thermal sensation scale (TS) [22]. A non-contact infrared thermometer (Data-Therm IR JXB-182) measured T_sk_ at the following sites: nape, forehead, sternum, biceps brachii of the playing arm, rectus femoris, and gastrocnemius [23], where the leg matching the playing arm was used. This enabled calculations for mean skin temperature (T_sk_) using Ramanathan’s [24] equation. The use of an infrared thermometer was validated by comparing measurements against skin thermistors during pilot testing. The limits of agreement between the infrared thermometer and skin thermistors determined by a Bland-Altman plot were 0.03 ± 3.14 °C. There was also a significant positive correlation between skin temperature measurements using the different methods (Grant Squirrel; *r* = 0.88). Following baseline measures, a Stroop test was conducted to measure cognitive reaction time [25], which consisted of 40 different stimuli, presented consecutively one after the other. Twenty were congruent (the word naming its colour) and 20 were incongruent (where the word and colour conflict). Players had to identify the colour of the stimulus regardless of the word that was represented. Players received a score out of 40 and a mean reaction time (that represents the accumulated total reaction time, divided by the number of correct answers). The experiment was programmed in E-Prime 2.08. The participants then wore a portable gas analyser (Cosmed K4B^2^, Cosmed, Rome, Italy), which recorded oxygen consumption ($\dot{V}$O_2_) throughout the warm up and exercise bouts. Participants then conducted their usual pre-match warm-up for 4 minutes against a mechanical ball thrower (robot) (Tibhar RoboPro Plus, TIBHAR Tibor Harangozo GmbH, Saarbrücken, Germany) using their own bats. An International Table Tennis Federation standard table (Butterfly Octet 25, Butterfly, Tokyo, Japan) was used, with 40 mm celluloid balls (Butterfly Star 3, Butterfly, Tokyo, Japan). Immediately following the warm-up, T_sk_, HR and TS were recorded. Participants rested for 2 minutes, however, during the final minute pre-cooling was applied during ICE. Cooling was applied during the rest periods between the three bouts during ICE, compared to no cooling in the control condition. T_sk_, HR and TS were measured immediately before and after each exercise bout and 5 minutes after the table tennis test ended, with ratings of perceived exertion (RPE) recorded only after each bout. Immediately following the final bout of exercise the Stroop test was conducted again.

#### 2.3.3. Table Tennis Specific Protocol

The present study implemented an exercise protocol simulating aspects of match play as recommended by Reilly et al. [26], and was designed to fatigue participants whilst lasting approximately 4 min, similar to real playing time in table tennis [27]. Participants completed three bouts, as this was the minimum number required to be completed for a best of five games match, a format the current participants normally compete in. Rest periods lasting 1-min (passive standing) were incorporated between bouts. The first bout consisted of forehand topspin shots only, where frequency was set at ≈45 balls·min^−1^ (robot setting 2) and speed on robot setting 5. Bout two comprised of backhand topspin shots only where the robot fed balls randomly but landed 0.5–0.6 m from the net and only within the backhand side of the table. After a pilot study, speed remained unchanged for bout 2, however, the frequency was increased to ≈53 balls·min^−1^ (setting 3) to closer match exercise intensities of bouts 1 and 3 due to the lower intensity of backhand shots. The third bout was identical to the first, except participants executed forehand and backhand topspins alternately. The robot threw balls randomly, however for bouts 1 and 3, balls landed 0.3–0.4 m either side of the centre line and 0.5–0.6 m from the net [28], enabling cross-table and down-the-line shots. Performance was measured by counting the number of times the intended target was hit within the first 80 shots played for bouts 1 and 3, and 90 shots for bout 2. A tripod mounted camcorder (Casio EX-FH100, Casio, Tokyo, Japan) verified ball landing spots filmed at 240 Hz. The first target, from the player’s viewpoint was left of the centre line, measuring 0.8 m from the net to the end of the table. The second target was the same except right of centre (Figure 1B). Both target area dimensions were 0.76 × 0.57 m. Participants alternately aimed for both targets throughout each bout and were given a performance score depending on how many shots they hit on target.

### 2.4. Statistical Analyses

SPSS 22.0 (IBM, New York City, NY, USA) was used to analyse data where mean ±standard deviations (±SD) are stated. Data normality was checked using a Shapiro-Wilk test and all data were found to be normally distributed. Statistical significance was set at *p* < 0.05. Post-hoc power analysis showed that the sample size used provided greater than 80% statistical power for the performance interaction between condition and bouts.

A within-group, 2-way (time × condition), repeated-measures analysis of variance (ANOVA) with post-hoc Bonferroni’s adjustment measured differences of T_sk_, HR, RPE and TS between conditions, whilst performance scores were measured for each corresponding bout between conditions (3 × 2). Partial Eta-Squared (η_p_^2^) was used to report effect size for ANOVA where effects were classified as small (0.01–0.05), moderate (0.06–0.13), and large (>0.14) [29]. Multiple paired-samples *t*-test using a Bonferroni correction measured differences of means for physiological variables and total performance score between conditions. Cohen’s *d* effect size was used for paired-samples *t*-test where effects were classified as small (0–0.2), moderate (0.3–0.79), and high (≥0.8) [29]. A paired-samples *t*-test was performed on the overall performance scores for trial 1 and trial 2 (irrelevant of cooling) determining that there was no order effect (*t*_(7)_ = −0.892; *p* = 0.40).

## 3. Results

### 3.1. Performance

**Figure 2:**
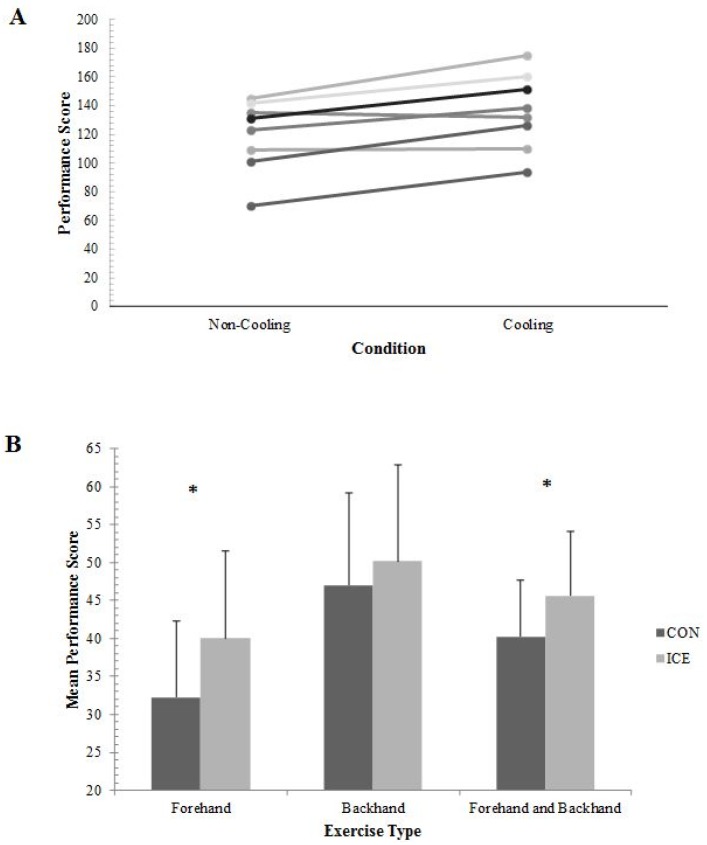
(**A**) Individual performance scores for both trials (**B**) Comparison of mean (±SD) performance scores between conditions for individual exercise bouts. * Denotes significant difference (*p* < 0.05) between conditions.

A main effect for condition (F_(1,7)_ = 15.61; P = 0.006, η_p_^2^ = 0.69) was observed, with seven out of eight participants improving overall performance in the ICE (136 ± 26 shots; Figure 2A) condition compared to CON (120 ± 25 shots; *p* = 0.006). There was a significant interaction between conditions and bouts (F_(2,14)_ = 4.09; *p* = 0.04, η_p_^2^ = 0.37). Figure 2B shows mean performance scores during ICE were greater than CON for both the forehand only bout (*t*_(7)_ = −7.65; *p* < 0.001, *d* = 0.72) and also the alternate forehand and backhand bout (*t*_(7)_ = −3.45; *p* = 0.011, *d* = 0.67). However, no difference (*t*_(7)_ = 1.43; *p* = 0.20, *d* = 0.25) was observed for backhand only.

### 3.2. Physiological Variables

**Figure 3:**
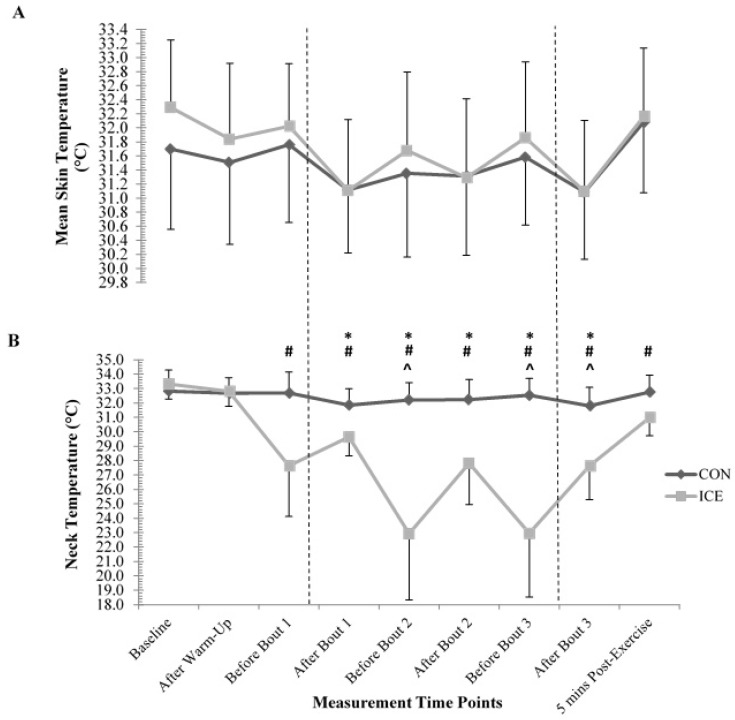
Mean ± SD of (**A**) mean skin temperature and (**B**) neck temperature measured during control (CON) and cooling (ICE) trials throughout testing procedure. * Denotes significant difference between conditions (*p* < 0.05) for neck temperature. ^#^ Denotes significant difference from ‘baseline’ (*p* < 0.05) for neck temperature. ^^^ Denotes significant difference from ‘5 min post-exercise’ (*p* < 0.05) for neck temperature. Vertical dashed lines are presented to clarify the periods during the testing procedure, pre-exercise, exercise protocol and post-exercise.

T_sk_ had a main effect for time (*p* < 0.001). Pairwise comparisons showed T_sk_ decreased during exercise and increased during recovery periods between bouts (Figure 3). There were no differences between trials for T_sk_ (*p* = 0.593). There was a significant interaction between condition and time at the T_neck_ site (F_(8,56)_ = 21.67; *p* < 0.001, η_p_^2^ = 0.756), with post-hoc analysis showing significantly lower T_neck_ after bout 1 until after bout 3 (*p* = 0.005 after bout 1, *p* = 0.009 after bout 2, and *p* = 0.002 after bout 3).

There were no differences in HR between conditions (F_(1,7)_ = 0.049; *p* = 0.831, η_p_^2^ = 0.007). There was a main effect for time (F_(2,14)_ = 35.51; *p* < 0.001, η_p_^2^ = 0.835) for $\dot{V}$O_2_, with post-hoc analysis showing $\dot{V}$O_2_, as well as HR, to be lower during bout 2 compared to bouts 1 and 3. The intensities of each bout were 80.0% ± 0.1%, 55.9% ± 0.1%, and 73.1% ± 0.2% $\dot{V}$O_2peak_ for bouts 1, 2, and 3, respectively. There was no main effect for condition (F_(1,7)_ = 0.00; *p* = 0.96, η_p_^2^ = 0) and no interaction between bout and condition (F_(2,14)_ = 0.35; *p* = 0.71, η_p_^2^ = 0.048).

### 3.3. Psychological Variables

Table 1 shows that there was a significant main effect for time (F_(8,56)_ = 12.67; *p* < 0.001, η_p_^2^ = 0.644), where TS increased after bout 1 and 3 compared to before each corresponding bout (*p* < 0.05). There was a significant main effect for condition with TS being significantly lower during ICE compared to CON (F_(1,7)_ = 7.67; *p* = 0.028, η_p_^2^ = 0.523).

RPE was significantly greater (*p* = 0.037) ‘after bout 1’ compared to ‘after bout 2’ (main effect for time; F_(2,14)_ = 9.32; *p* = 0.003, η_p_^2^ = 0.571). RPE was highest ‘after bout 1’, lowest ‘after bout 2’, and moderate ‘after bout 3’ for both conditions (Table 1). There was no main effect for condition (F_(1,7)_ = 0.15; *p* = 0.708, η_p_^2^ = 0.021).

Stroop test accuracy showed no interaction or main effects for time and condition (*p* > 0.05). No interaction was observed (F_(1,7)_ = 0.006; *p* = 0.94, η_p_^2^ = 0.001) for Stroop reaction time, however, a main effect for time was seen (F_(1,7)_ = 37.38; *p* < 0.001, η_p_^2^ = 0.842) where quicker times were recorded post-exercise (Table 1). 

### 3.4. Correlations

There was a significant, moderate negative correlation between RPE and individual bout performance score (*p* < 0.001; *r* = −0.53), demonstrating that as RPE decreased, performance improved. There was also a significant moderate correlation between RPE and TS (*p* < 0.001; *r* = 0.62), demonstrating that as RPE increased, so did TS. Thirdly, a significant moderate correlation between HR and TS (*p* < 0.001; *r* = 0.38) was found, indicating HR and TS increased concurrently. Finally, measures of exercise intensity were comparable, as indicated by a significant moderate correlation between $\dot{V}$O_2_ and HR during exercise (*p* < 0.001; *r* = 0.54).

## 4. Discussion

The primary aim of the study was to examine the effects of intermittent cooling on a table tennis-specific performance test and assess the concomitant thermoregulatory responses. It was hypothesised that cooling would improve table tennis performance by lowering RPE and thermal sensation as opposed to inducing significant physiological changes. The most important finding suggests the hypothesis was partially correct as cooling significantly improved table tennis performance, on average by 16 shots on target compared to the control trial. Thermal sensation significantly reduced with cooling, however, RPE was similar between conditions, thereby suggesting that cooling did not significantly reduce RPE.

This study attempted to induce similar match play fatigue levels through a simulated table-tennis specific protocol. The players in the present study performed at an average exercise intensity of 65% ± 8% of HR_max_. This value is similar to those of Suchomel [30] who studied older players (23.6–24.4 years), but due to the data being relative to their maximum HR this justifies comparisons. Therefore, the similar data suggests that the protocol induced match play exercise intensity and reflected fatigue induced during table tennis match play. Fatigue has been demonstrated to affect skill performance [31]. In particular, central fatigue impairs cognition, neuromuscular function, and consequently performance in racquet sports [32]. This study assessed cognition and neuromuscular reactivity through Stroop tests, but found no significant differences between conditions for Stroop test accuracy or reaction time. This suggests cooling did not affect cognition or neuromuscular reactivity, as this specific table tennis skills protocol may not have induced sufficient fatigue. Previous research found no differences in cognitive function with cooling [33] and neuromuscular reactivity [3] after simulated tennis and table tennis protocols, respectively.

Despite recurring patterns throughout testing for physiological and psychological variables, differences between conditions were insignificant for all variables except TS and neck temperature. During the cooling trial, neck temperature was significantly lower throughout testing, showing only the site of application was affected by cooling, concurring with Tyler and Sunderland [15]. Neck cooling significantly reduced thermal sensation and therefore reduced the perception of whole-body temperature. Tyler and Sunderland [15] found neck cooling decreased thermal sensation and perceived fatigue, thereby leading to improved performance, potentially due to lower brain temperature. However, in this study, cooling probably did not further reduce brain temperature, as core and forehead temperatures were similar between conditions. Conversely, as the face and neck are regions of high alliesthesial thermosensitivity [16], cooling probably reduced perceived thermal sensation in this study by disguising the magnitude of thermal stress experienced.

There was a negative correlation between RPE and individual performance scores, suggesting performance improved with lower RPE. HR and RPE were positively correlated to thermal sensation, suggesting RPE and potentially perceived fatigue were greater when thermal sensation was elevated. Seven out of eight participants reported lower thermal sensation with cooling, which probably caused the central governor to perceive lower body temperature, fatigue, and exertion, thus enabling maintenance of higher exercise intensities and better-quality neuromuscular function [13,34]. Consequently, fine motor-skills are sustained through superior muscle recruitment, leading to performance improvements compared to the control trial [35]. This central fatigue theory partially explains the mechanism for performance improvements seen with local cooling during short-term high-intensity exercise, also suggested by Tyler and Sunderland [15] and Kwon, Robergs and Schneider [35]. However, further research is warranted, particularly within skill-based sports, to confirm the causal relationship. Performance improvements with cooling in the present study were greatest in higher intensity exercises, suggesting the effectiveness of cooling upon the central governor intensified with increased perceptions of effort. This is supported by results which demonstrated the higher intensity forehand bout (80.0% ± 0.1% $\dot{V}$O_2peak_) to have a greater improvement in performance than the lower intensity backhand bout (55.9% ± 0.1% $\dot{V}$O_2peak_). Players may therefore apply neck cooling to improve performance particularly when intensities are elevated.

The inclusion of a neck cooling intervention was to provide an immediate, short-term improvement in table tennis performance, therefore subsequent adaptations to cooling were not considered to be of primary importance. Notwithstanding, there is a paucity of research regarding the effect of cooling interventions on the development of thermoregulatory adaptations in sport. Reductions in heart rate, sweat rate, skin blood flow, plasma volume, and a slower rise in T_c_ are physiological thermoregulatory adaptations [36] which may be prevented by the application of a cooling intervention. Adaptations to thermoreceptors also occur upon external cooling, where the number of sensitive cold receptors decrease and the cold sensation threshold increases, resulting in increased difficulty in sensing colder temperatures [37]. This follows that psychologically, participants may become accustomed to the cooling intervention upon chronic application, therefore potentially reducing the efficacy of the intervention during subsequent bouts of application. However, this remains inconclusive and longitudinal research studies are required to ascertain whether familiarisation to cooling interventions affects performance. Consequently, cooling interventions should be used at times when athletes are under high thermal stress which would normally occur after an intensive bout of exercise or towards the latter part of the match, therefore neck cooling does not need to be applied during all interval periods. Individuals that are particularly susceptible to thermal stress and those who perceive neck cooling benefits their performance are encouraged to use the intervention.

Previous studies [15,16] involving cooling interventions showed performance improvements in high ambient conditions. The findings from the present study suggest that table tennis performance improved due to the positive psychological impact of neck cooling. Therefore, players and coaches are encouraged to use this intervention during training sessions and/or match play under moderate and high ambient conditions. It is advised that players should undergo a period of familiarisation with neck cooling prior to consistent use in training or competition in order to ascertain their individual physiological, perceptual, and performance responses to cooling.

## 5. Conclusions

In conclusion, this study provides novel findings suggesting intermittent neck cooling improves simulated table tennis performance by lowering thermal sensation and potentially impacting the central governor into perceiving less fatigue. This is supported by performance improvements of 16 ± 12 shots on target during ICE compared to CON. Further research is required to understand the effects of intermittent neck cooling on table tennis performance during actual match play and the concomitant thermoregulatory responses. However, the application of this intervention during training may improve the quality of practice, thus having a beneficial knock-on effect on match performance.

## Figures and Tables

**Table 1:** Mean (±SD) of physiological and psychological variables during CON and ICE trials throughout the testing procedure. * Significant difference between conditions for respective measures, P < 0.05. **^** Significant difference between conditions for respective measure, P < 0.01.

		Pre-Test	Baseline	After Warm-Up	Before Bout 1	After Bout 1	Before Bout 2	After Bout 2	Before Bout 3	After Bout 3	5 min Post-Exercise	Post-Test
HR (bpm)	CON		91.50 ± 17.53	132.75 ± 23.70	98.88 ± 23.37	171.25 ± 14.85	109.38 ± 20.93	139 ± 21.17	105.5 ± 16.12	162.63 ± 25.13	102.25 ± 16.46	
	ICE		84.25 ± 7.70	132.5 ± 23.53	96.13 ± 12.17	172.75 ± 16.88	108.13 ± 8.46	151.38 ± 20.69	106.63 ± 12.16	167.63 ± 22.47	101.63 ± 7.15	
VO_2_ (L·min^−1^)	CON					2.54 ± 0.41		1.78 ± 0.42		2.35 ± 0.49		
	ICE					2.58 ± 0.25		1.81 ± 0.34		2.29 ± 0.45		
TS (AU)	CON		4.38 ± 0.74	5.25 ± 1.04 *	5 ± 1.2 *	6.13 ± 0.64	5.25 ± 1.04	5.75 ± 1.16	5.13 ± 1.36 *	6.25 ± 0.89 **^**	4.88 ± 1.13	
	ICE		4.13 ± 0.83	4.63 ± 1.06	3.5 ± 1.51	5.75 ± 0.46	4.5 ± 1.6	5.38 ± 0.92	4.5 ± 1.31	5.38 ± 0.92	4.5 ± 0.76	
RPE (AU)	CON					16.13 ± 1.46		12.5 ± 2.78		14.5 ± 1.85		
	ICE					15.25 ± 1.49		13.25 ± 2.31		14.25 ± 1.49		
Stroop Accuracy (AU)	CON	37.75 ± 1.67										37.63 ± 1.6
	ICE	38.16 ± 1.81										37.63 ± 1.92
Stroop Reaction Time (ms)	CON	646.99 ± 106.54										578.66 ± 104.2
	ICE	617.38 ± 142.33										545.77 ± 137.92
